# Construction of a broad-host-range Anderson promoter series and particulate methane monooxygenase promoter variants expand the methanotroph genetic toolbox

**DOI:** 10.1016/j.synbio.2024.02.003

**Published:** 2024-02-19

**Authors:** Etash H. Bhat, Jessica M. Henard, Spencer A. Lee, Dustin McHalffey, Mahith S. Ravulapati, Elle V. Rogers, Logan Yu, David Skiles, Calvin A. Henard

**Affiliations:** Department of Biological Sciences and BioDiscovery Institute, University of North Texas, Denton, TX, USA

**Keywords:** Methanotroph, Methane monooxygenase, Promoter, Metabolic engineering, Synthetic biology

## Abstract

Methanotrophic bacteria are currently used industrially for the bioconversion of methane-rich natural gas and anaerobic digestion-derived biogas to valuable products. These bacteria may also serve to mitigate the negative effects of climate change by capturing atmospheric greenhouse gases. Several genetic tools have previously been developed for genetic and metabolic engineering of methanotrophs. However, the available tools for use in methanotrophs are significantly underdeveloped compared to many other industrially relevant bacteria, which hinders genetic and metabolic engineering of these biocatalysts. As such, expansion of the methanotroph genetic toolbox is needed to further our understanding of methanotrophy and develop biotechnologies that leverage these unique microbes for mitigation and conversion of methane to valuable products. Here, we determined the copy number of three broad-host-range plasmids in *Methylococcus capsulatus* Bath and *Methylosinus trichosporium* OB3b, representing phylogenetically diverse Gammaproteobacterial and Alphaproteobacterial methanotrophs, respectively. Further, we show that the commonly used synthetic Anderson series promoters are functional and exhibit similar relative activity in *M. capsulatus* and *M. trichosporium* OB3b, but the synthetic series had limited range. Thus, we mutagenized the native *M. capsulatus* particulate methane monooxygenase promoter and identified variants with activity that expand the activity range of synthetic, constitutive promoters functional not only in *M. capsulatus*, but also in *Escherichia coli*. Collectively, the tools developed here advance the methanotroph genetic engineering toolbox and represent additional synthetic genetic parts that may have broad applicability in Pseudomonadota bacteria.

## 1 Introduction

Methane (CH_4_) is the primary component of natural gas and biogas and the second-most abundant greenhouse gas (GHG) in the atmosphere, contributing roughly 25 percent towards the elevated temperature associated with climate change [1]. A potential route to mitigate GHGs is through the biological conversion of CH_4_ by methanotrophic bacteria (methanotrophs). Methanotrophs have the unique ability to utilize CH_4_ as a carbon and energy source, activating the C–H bond at ambient temperature and pressure using the enzyme methane monooxygenase that is unique to this group of microbes. CH_4_ represents a sustainable carbon source for industrial manufacturing and its conversion by methanotrophs would not only decrease GHGs, but also valorize squandered single-carbon sources, such as those that are currently flared or uncaptured.

The pressing need to decrease atmospheric CH_4_ levels and develop sustainable biotechnologies has resulted in significant advances in understanding fundamental aspects of methanotroph metabolism and development of genetic tools for use in these bacteria. Several broad-host-range (BHR) replicative and non-replicating suicide plasmids capable of conjugal transfer from *Escherichia coli* to proteobacterial methanotrophs have enabled reverse genetic approaches to determine gene-function relationships in these bacteria [[2], [3], [4], [5], [6]]. Incompatibility group P (IncP) BHR plasmids have been the primary backbone for the development of expression plasmids with regulatory DNA elements functional in phylogenetically diverse methanotrophs [4,7,8]. A regulatory element central to controlling transcription of native or heterologous genes is the promoter element recognized by the RNA polymerase holoenzyme. The strength of the binding interaction between the RNA polymerase holoenzyme and the promoter sequence, which can be modulated by transcription factors, is positively correlated to transcription initiation [9]; thus, RNA polymerase has high affinity for “strong” promoters and low affinity for “weak” promoters.

In methanotrophs, native promoters associated with the most highly expressed genes, such as the particulate methane monooxygenase operon promoter (P_*pmoC*_) and the calcium-dependent methanol dehydrogenase operon promoter (P_*mxaF*_), have been leveraged to drive transcription from expression vectors in Gammaproteobacteria and Alphaproteobacteria methanotrophs [4,10]. Additionally, the commonly used *E. coli* P_*tac*_ promoter exhibits comparable activity compared to the native “strong” promoters in *Methylotuvimicrobium* [4,11,12], *Methylococcus* [10,13], and *Methylomonas* [14]*.* Several inducible promoter systems reliant on allosterically regulated transcriptional regulators (e.g. TetR, AraC) have also been shown to function in industrially relevant methanotrophs [[15], [16], [17]], which have enabled advanced methanotroph gene editing technologies like CRISPR-Cas to be developed [10,18,19]. Collectively, BHR plasmids and regulatory genetic parts have been used to express native, heterologous, and synthetic biochemical pathways in engineered methanotrophs to produce valuable molecules directly from CH_4_ [6,20] and references therein). However, the current parts in the methanotroph genetic toolbox are lacking the characterization required for fine-tuned gene expression in CH_4_ biocatalysts, and expansion of the toolbox is needed to advance methanotroph research and development.

In this study, we quantified the copy number of the commonly utilized BHR plasmids pCAH01 (IncP), pQCH (IncQ), and pBMTL-2 (pBBR1) in the Gammaproteobacterial methanotroph *Methylococcus capsulatus* Bath and the Alphaproteobacterial methanotroph *Methylosinus trichosporium* OB3b. Using these BHR plasmids to construct new expression vectors, we compared the Anderson series promoters from the Registry of Standard Biology Parts in both *M. capsulatus* and *M. trichosporium*. Further, we mutagenized the *M. capsulatus* particulate methane monooxygenase promoter (P_*pmoc2*_) and isolated variants with activity that increase the dynamic range of promoter activity in methanotrophs. These developments expand the methanotroph genetic toolbox that can be used to easily and precisely engineer these bacteria to convert CH_4_ to high-value compounds and mitigate atmospheric CH_4_.

## 2 Materials and methods

### 2.1 Bacterial cultivation

Bacterial strains used in this study are shown in Table 1. DH10b and S17-1λpir *E*. *coli* were cultured in lysogeny broth (Lennox) with 50 μg/mL kanamycin or 10 μg/mL gentamicin for transformant selection. *M*. *capsulatus* Bath and *M*. *trichosporium* OB3b cultures were routinely maintained with nitrate mineral salts (NMS) solid medium in stainless steel gas chambers supplied with 20% CH_4_ in the gas phase at 37 °C or 30 °C, respectively, as previously described [10]. Plasmids were transferred to methanotrophs via biparental mating by spreading equivalent biomass of S17-1λ *E. coli* and recipient methanotroph biomass on NMS mating agar mating plates and incubating in a 20% CH_4_ atmosphere for 24 h as previously described [10]. Methanotroph transformants harboring plasmids pCAH01, pQCH, pBMTL-2, or pMMO promoter expression plasmids were selected on NMS medium containing 50 μg/mL kanamycin while transformants harboring pBBR1MCS-5 Anderson series plasmids were selected on NMS medium containing 10 μg/mL gentamicin. Methanotrophs were also cultured in 150 mL vials containing 10 mL of NMS medium at 37°C (*M. capsulatus*) or 30°C (*M. trichosporium*) at 200 rpm orbital shaking. After inoculation with plate-derived biomass to OD_600_ = 0.1, vials were crimped with grey butyl stoppers to create gas-tight seals followed by CH_4_ addition to the headspace via syringe to reach a final CH_4_ concentration of 20% in air (v/v). Cultures were incubated with orbital shaking for 24 h with appropriate antibiotics prior to DNA extraction or fluorescence measurement.

**Table 1:** Strains and plasmids.

Name	Genotype	Source
***Methylococcus capsulatus* str. Bath**	Wild-type	ATCC 33009
***Methylosinus trichosporium* str. OB3b**	Wild-type	[21]
***Escherichia coli* str. Zymo 10B**	F- *mcrA* Δ(*mrr*-*hsdRMS*-*mcrBC*) Φ80*lacZ*ΔM15 Δ*lacX*74 *recA1 endA1 araD139* Δ(*ara leu*) 7697 *galU galK rpsL nupG*	Zymo Research
***E. coli* S17**–**1**	Tp^r^ Sm^r^*recA thi pro hsd* (r^-^m^+^)RP4-2-Tc:Mu:Km Tn7	ATCC 47055

Plasmids		
Name	Description	Source
**pCAH01**	IncP BHR inducible expression plasmid	[15]
**pQCH**	IncQ BHR plasmid	[10]
**pBMTL-2**	pBBR1 BHR plasmid	[22]
**pBBR1MCS-5**	Plasmid backbone for Anderson series; Gm^R^	[23]
**pDS1**	pBBR1MCS-5 with BBa_J23119-mRFP1 reporter	This study
**pAS100**	pBBR1MCS-5 with BBa_J23100-mRFP1 reporter	This study
**pAS101**	pBBR1MCS-5 with BBa_J23101-mRFP1 reporter	This study
**pAS102**	pBBR1MCS-5 with BBa_J23102-mRFP1 reporter	This study
**pAS104**	pBBR1MCS-5 with BBa_J23104-mRFP1 reporter	This study
**pAS105**	pBBR1MCS-5 with BBa_J23105-mRFP1 reporter	This study
**pAS106**	pBBR1MCS-5 with BBa_J23106-mRFP1 reporter	This study
**pAS107**	pBBR1MCS-5 with BBa_J23107-mRFP1 reporter	This study
**pAS110**	pBBR1MCS-5 with BBa_J23110-mRFP1 reporter	This study
**pAS114**	pBBR1MCS-5 with BBa_J23114-mRFP1 reporter	This study
**pAS115**	pBBR1MCS-5 with BBa_J23115-mRFP1 reporter	This study
**pAS116**	pBBR1MCS-5 with BBa_J23116-mRFP1 reporter	This study
**pAS117**	pBBR1MCS-5 with BBa_J23117-mRFP1 reporter	This study
**pAS118**	pBBR1MCS-5 with BBa_J23118-mRFP1 reporter	This study
**pJH1**	pBBR1MCS-5 with BBa_J23119-sfgfp reporter	This study
**pQCHP**_***pmoC2***_**-sfgfp**	pQCH with the MCA2855 promoter driving *sfgfp* expression	[10]
**pHSP**_***6***_**-sfgfp**	pQCH with the MCA2855 P_*pmoC2*_ promoter variant (−35T to A) driving *sfgfp* expression	This study
**pHSP**_***8***_**-sfgfp**	pQCH with the MCA2855 P_*pmoC2*_ promoter variant (−31C to T and -3C to T) driving *sfgfp* expression	This study
**pHSP**_***11***_**-sfgfp**	pQCH with the MCA2855 P_*pmoC2*_ promoter variant (−43G to A)driving *sfgfp* expression	This study
**pHSP**_***14***_**-sfgfp**	pQCH with the MCA2855 P_*pmoC2*_ promoter variant (−43G to T) driving *sfgfp* expression	This study
**pHSP**_***16***_**-sfgfp**	pQCH with the MCA2855 P_*pmoC2*_ promoter variant (−35T to C and -9C to A) driving *sfgfp* expression	This study
**pJH2**	pQCH with BBa_J23119*-sfgfp*	This study

### 2.2 Plasmid copy number determination

Genomic DNA was extracted from 1 mL (∼1e^7^ cfu/mL) methanotrophic bacteria cultured 24 h (starting OD_600_ = 0.1) in liquid NMS with 25 μg/mL kanamycin using the DNeasy Blood and Tissue DNA extraction kit following the manufacturer's protocol (Qiagen). 10 ng gDNA from three independent transformants for each plasmid was used as template for quantitative PCR using a primer set targeting the plasmid *ahp* kanamycin resistance gene (oCAH956/957) or the single-copy chromosomal *rpoB* gene of *M. capsulatus* or *M. trichosporium* (oCAH906/907 or oCAH910/911, respectively) that encodes the β subunit of RNA polymerase (Table 2). Primer sets were confirmed to have similar efficiencies (>98%) using a dilution series of purified gDNA. Copy number (CN) was determined by relative comparison of the cycle threshold (Ct) values for each target using the following equation: CN = 2^−Ct*ahp*-Ct*rpoB*^ [24,25].

**Table 2:** Primers and synthetic DNA fragments.

Name	Sequence
***Plasmid copy number determination***	
**oCAH906 Bath *rpoB* F**	GCCAAGGTGAATCAGGAGAT
**oCAH907 Bath *rpoB* R**	GGTCGAGATCGTTCACATAGAG
**oCAH910 OB3b *rpoB* F**	CAAATCCGTCTTCCCGATCTC
**oCAH911 OB3b *rpoB* R**	GCACTCGTCGACGTCATATT
**oCAH956 *ahp*/kn F**	TGCGCCAGAGTTGTTTCT
**oCAH957 *ahp*/kn R**	GATGGTCGGAAGAGGCATAAA
***Construction of pDS1 and BHR Anderson series promoter-probe plasmids***	
**mRFP1 reporter**	gcaatagacataagcggctaGCCCTCTAGAGGTGCAAAACCTTTCGCGGTATGGCATGATAGCGCCCGGAAGAGAGTCAATTCAGGGTGGTGAATTTGACAGCTAGCTCAGTCCTAGGTATAATAGATCTGAATTCATTAAAGAGGAGAAAGGTACCATGGCGAGTAGCGAAGACGTTATCAAAGAGTTCATGCGTTTCAAAGTTCGTATGGAAGGTTCCGTTAACGGTCACGAGTTCGAAATCGAAGGTGAAGGTGAAGGTCGTCCGTACGAAGGTACCCAGACCGCTAAACTGAAAGTTACCAAAGGTGGTCCGCTGCCGTTCGCTTGGGACATCCTGTCCCCGCAGTTCCAGTACGGTTCCAAAGCTTACGTTAAACACCCGGCTGACATCCCGGACTACCTGAAACTGTCCTTCCCGGAAGGTTTCAAATGGGAACGTGTTATGAACTTCGAAGACGGTGGTGTTGTTACCGTTACCCAGGACTCCTCCCTGCAAGACGGTGAGTTCATCTACAAAGTTAAACTGCGTGGTACCAACTTCCCGTCCGACGGTCCGGTTATGCAGAAAAAAACCATGGGTTGGGAAGCTTCCACCGAACGTATGTACCCGGAAGACGGTGCTCTGAAAGGTGAAATCAAAATGCGTCTGAAACTGAAAGACGGTGGTCACTACGACGCTGAAGTTAAAACCACCTACATGGCTAAAAAACCGGTTCAGCTGCCGGGTGCTTACAAAACCGACATCAAACTGGACATCACCTCCCACAACGAAGACTACACCATCGTTGAACAGTACGAACGTGCTGAAGGTCGTCACTCCACCGGTGCTTAAGGATCCAAACTCGAGTAAGGATCTCCAGGCATCAAATAAAACGAAAGGCTCAGTCGAAAGACTGGGCCTTTCGTTTTATCTGTTGTTTGTCGGTGAACGCTCTCTACTAGAGTCACACTGGCTCACCTTCGGGTGGGCCTTTCTGCGTTTATAtcactatagggcgaattgga
**oCAH16 pBBR R**	TAGCCGCTTATGTCTATTGCTG
**oCAH17 pBBR F**	TCACTATAGGGCGAATTGGAG
**oCAH1194 Anderson F**	gcaatagacataagcggctaTCGCTAAGGATGATTTCTGGAATTC
**oCAH1195 Anderson R**	ccttactcgagtttggatccTTAAGCACCGGTGGAGTG
**oCAH1196 pDS1 F**	GGATCCAAACTCGAGTAAGGATC
***P***_***pmoc2***_***promoter mutagenesis***	
**oCAH1303 pQCHPpmoC2 F**	CGTGGGCGCGGCTCTGAG
**oCAH1304 pQCHPpmoC2 R**	GCCGGGCACTTGGATGAAAAAGAGA
**oCAH1305 PpmoC2mut F**	TCTCTTTTTCATCCAAGTGCCCGGC
**oCAH1306 PpmoC2mut R**	CTCAGAGCCGCGCCCACG
***Construction of pJH1 and pJH2***	
**oCAH1008 pDS1 R**	GGTACCTTTCTCCTCTTTAATG
**oCAH1009 *sfgfp* F**	attcattaaagaggagaaaggtaccATGAGCAAAGGAGAAGAAC
**oCAH1010 *sfgfp* R**	gagatccttactcgagtttggatccTTATTTGTAGAGCTCATCC
**oCAH28 pQCH F**	ATAAAACGAAAGGCTCAGTC
**oCAH190 pQCH R**	TATTGCAAGGACGCGGAAC
**oCAH1326 BBa_J23119*-sfgfp* F**	aggcatgttccgcgtccttgcaataAACCTTTCGCGGTATGGCAT
**oCAH1327 BBa_J23119*-sfgfp* R**	gactgagcctttcgttttatTTATTTGTAGAGCTCATCCATGCCA

Lowercase sequence are homology arms for isothermal assembly.

### 2.3 BHR Anderson promoter series construction and relative activity measurement

A DNA fragment consisting of BBa_J23119 promoter-Bujard RBS-mRFP1-BBa_B00015 terminator (termed mRFP1 reporter, Table 2) was designed using parts from the Repository of Standard Biological Parts (http://parts.igem.org) and synthesized by Integrated DNA Technologies. Plasmid pBBRMCS1-5 was amplified with primers oCAH16 and oCAH17 and assembled with the synthetic mRFP1 reporter fragment using HiFi Gibson Assembly Master Mix (New England Biolabs) to generate the plasmid pDS1. DNA fragments containing an Anderson series promoter-Bujard RBS-mRFP1 were amplified from BBa_J61002 supplied with the iGEM 2021 distribution kit using primers oCAH1194 and oCAH1195 and assembled with pDS1 amplified with primers oCAH1196 and oCAH16 to generate a BHR Anderson promoter-probe plasmid series. Notably promoter parts BBa_J23103, BBa_J23108, BBa_J23109, BBa_J23111, BBa_J23112 were not constructed here either because they exhibit limited activity in *E. coli* or they exhibit redundant activity with other promoters in the series. *E. coli* DH10B, *M. capsulatus* Bath, or *M. trichosporium* OB3b harboring the Anderson promoter-probe series were cultivated in liquid medium containing 10 μg/mL gentamicin to ∼ OD_600_ 1.0; 200 μL culture was transferred to a 96-well microplate, and mRFP1 fluorescence (ex_532nm_, em_588nm_, gain = 80) and optical density (A_600nm_) was measured with a BioTek Synergy Mx microplate reader.

### 2.4 Particulate methane monooxygenase promoter mutagenesis and screening

The *M. capsulatus* Bath particulate methane monooxygenase subunit C gene (*pmoC2*; MCA2855) promoter region spanning −113 to +37 that includes the putative UP, −35, −10 promoter elements, and ∼50 bp upstream and downstream of these elements was amplified from purified genomic DNA using primers oCAH1305 and oCAH1306. The 150 bp P_*pmoC2*_ amplicon (40 ng) was used as template for random mutagenesis with the GeneMorph II Random Mutagenesis kit (Agilent) and the primers used to amplify the template following the manufacturer's recommended parameters with an annealing temperature of 54 °C and 30 cycles. The mutagenized amplicon was assembled with the previously developed pQCHP_*pmoC2*_-*sfgfp* promoter-probe plasmid [10] amplified with primers oCAH1303 and oCAH1304. Plasmids isolated from five randomly chosen *E. coli* DH10B transformants selected on LB agar containing 50 μg/mL kanamycin were sequenced to determine the mutation frequency prior to additional screening. To facilitate comparison between the P_*pmoC2*_ variant activity and the Anderson series promoter activity, the pJH1 promoter-probe plasmid was constructed by replacing mRFP1 in pDS1 with the *sfgfp* gene encoding superfolder GFP (sfGFP) using primers oCAH1009 and oCAH1010 to amplify *sfgfp* and oCAH1196 and oCAH1008 to linearize pDS1. The P_*pmoc2*_*-sfgfp* region of pQCHP_*pmoC2*_-*sfgfp* amplified with oCAH28 and oCAH190 was replaced with BBa_J23119*-sfgfp* PCR-amplified from pJH1 with primers oCAH1326 and oCAH1327 to generate pJH2. *E. coli* DH10B and *M. capsulatus* Bath harboring the P_*pmoC2*_ promoter variant series were cultivated in liquid medium containing 25 μg/mL kanamycin to ∼ OD_600_ 1.0; 200 μL culture was transferred to a 96-well microplate, and sfGFP fluorescence (ex_465nm_, em_510nm_, gain 50) and optical density (A_600nm_) was measured with a BioTek Synergy Mx microplate plate reader.

## 3 Results and discussion

### 3.1 Plasmid copy number varies between Gammaproteobacteria and Alphaproteobacteria methanotrophs

**Fig. 1:**
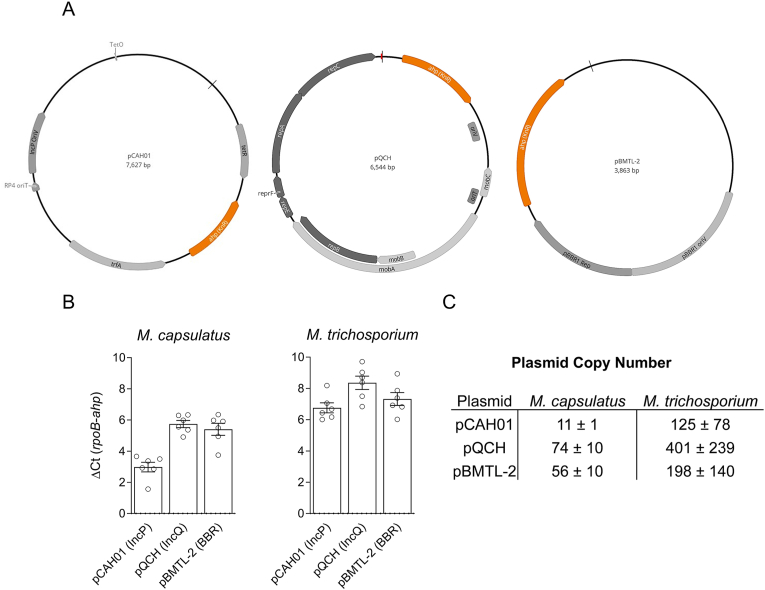
**Broad-host-range plasmid copy number in phylogenetically diverse methanotrophs.** A) Broad-host-range IncP- (pCAH01), IncQ- (pQCH), and pBBR-based (pBMTL-2) plasmid maps. Replicon and antibiotic resistance genes are highlighted in grey or orange, respectively. B) The cycle threshold (Ct) difference between the single copy RNA polymerase β subunit *rpoB* gene and the plasmid kanamycin resistance *ahp* gene in genomic DNA extracted from *M. capsulatus* or *M. trichosporium* plasmid-harboring transformants determined by quantitative PCR. C) Plasmid copy number calculated using qPCR data. The data in B and C represent the mean ± SEM from six individual transformants.

The plasmid copy number maintained by the host cell can have a significant impact on gene expression levels, enzyme production, and cellular fitness [26]; thus, plasmid copy number is an important consideration in metabolic engineering and synthetic biology using plasmid-based expression. Methanotrophs can replicate several BHR origins of replication, including IncP, IncQ, IncW, and pBBR plasmids [19], and derivatives thereof have been leveraged in the development of engineered methanotrophic biocatalysts [[27], [28], [29]]. However, the copy number of these BHR plasmids maintained by methanotrophs is unknown. We determined the copy number of plasmids from the most commonly used IncP-, IncQ-, and pBBR replicons, including pCAH01 (IncP), pQCH (IncQ), and pBMTL-2 (pBBR), in industrially relevant *M. capsulatus* Bath and *M. trichosporium* OB3b (Fig. 1A), representing phylogenetically diverse Gammaproteobacteria and Alphaproteobacteria methanotrophs. Primers targeting the plasmid kanamycin resistance *ahp* gene or the single-copy, chromosomal *rpoB* gene of *M. capsulatus* or *M. trichosporium* were designed and used to compare the relative plasmid copy number via quantitative PCR (Fig. 1B).

The IncP-based plasmid pCAH01 showed the lowest copy number (11 ± 1) in *M. capsulatus* Bath followed by pBMTL-2 (56 ± 10) and pQCH (74 ± 10) (Fig. 1C). All plasmids were maintained at higher copy number by *M. trichosporium* OB3b, although the relative trend observed in *M. capsulatus* was similar wherein pCAH01 was the lowest (125 ± 78), followed by pBMTL-2 (198 ± 140), and pQCH (401 ± 239). Notably, the high copy number of pQCH was correlated to an *M. trichosporium* OB3b growth defect as the appearance of transformants on selection plates was delayed (∼1 month until transformant colonies appeared) compared to pCAH01 and pBMTL-2 transformant colony formation (∼1 week). The higher plasmid copy number in *M. trichosporium* OB3b was unexpected since this bacterium maintains three native plasmids [21]. We observed greater copy number variation between *M. trichosporium* OB3b transformants compared to *M. capsulatus* (Fig. 1B and C), perhaps due to plasmid instability/competition with these three native plasmids. Although we determined copy number of pCAH01, pQCH, and pBMTL-2, we expect that other IncP-, IncQ-, and pBBR-based plasmids, including the commonly used IncP-based pAWP plasmids [4], are maintained at similar levels.

### 3.2 Construction of a BHR Anderson promoter series and characterization in Gamma- and Alphaproteobacterial methanotrophs

**Fig. 2:**
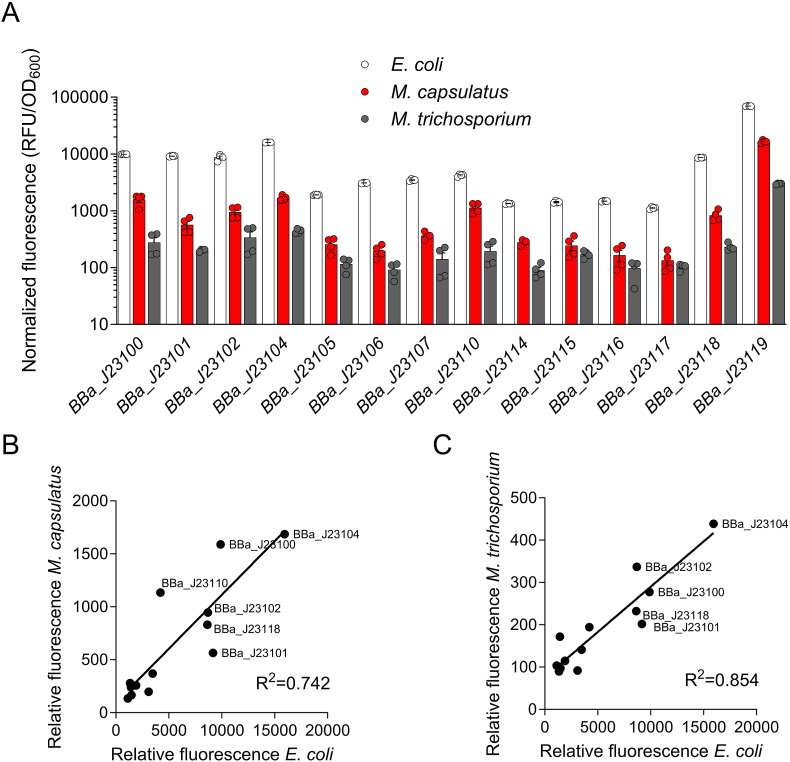
**Comparison of the Anderson series promoter activity in *E. coli* and diverse methanotrophs.** Relative Anderson series promoter activity in *E. coli* (white bar), *M. capsulatus* (red bar), and *M. trichosporium* (grey bar) determined by mRFP1 fluorescence during logarithmic growth phase cells. Linear regression analysis comparing relative Anderson series promoter activity in *E. coli* to that in *M. capsulatus* (B) or *M. trichosporium* (C). The data represent the mean ± SEM from two independent experiments (n = 4).

BHR plasmids have been used to develop both constitutive and inducible expression plasmids with heterologous *E. coli* P_*tac*_, P_*lac*_, P_*ara*_, P_*tet*_ and native promoters from highly expressed genes (e.g., methanol dehydrogenase P_*mxa*_) to control transcription in methanotrophs [4,10,[30], [31], [32], [33]]. However, quantitative assessments of these promoters are lacking and the methanotroph toolbox promoter repertoire, in general, is limited, hindering metabolic engineering efforts for fine-tuned transcriptional control in these microbes. To address this limitation, we evaluated the Anderson promoter series from the Registry of Biological Parts, which have been demonstrated to function in phylogenetically diverse bacteria [[34], [35], [36]]. The promoter collection represents a small combinatorial mutagenesis library of the *E. coli* core consensus promoter (part BBa_J23119). The parts from the Registry consist of an Anderson promoter (BBa_J23100-119) driving expression of the mRFP1 gene for fluorescence-based quantification of promoter activity, which are in the BBa_J61002 backbone supplied with the 2021 iGEM distribution kit. We transferred the BBa_J23(100–119)-mRFP1 cassettes from the BBa_J61002 plasmid to the pBBRMCS1-5 BHR plasmid to construct a BHR Anderson series collection for expression in both *E. coli* and methanotrophic bacteria. Notably promoter parts BBa_J23103, BBa_J23108, BBa_J23109 BBa_J23111, BBa_J23112 were not constructed here either because they exhibit limited activity in *E. coli* or they exhibit redundant activity with other promoters in the series. The promoter collection was transferred to *M. capsulatus* Bath or *M. trichosporium* OB3b via biparental mating and mRFP1 fluorescence was measured as a readout of promoter activity in the bacterial strains (Fig. 2A). The consensus promoter BBa_J23119 exhibited the highest activity in all strains tested. We observed BBa_J23104 to have the highest activity of the mutated series followed by BBa_J23100 in *E. coli* as well as both methanotrophs. Anderson series promoter activity was decreased in *M. capsulatus* Bath by 4- to 16-fold depending on the promoter compared to *E. coli*. Although functional as indicated by fluorescence compared to empty vector controls, promoter activity in *M. trichosporium* OB3b showed a 20- to 40-fold decrease compared to *E. coli*, consistent with substantial differences in the core promoter elements between Alphaproteobacteria and Gammaproteobacteria [37,38].

Regression analysis of the promoter collection activity in *M. capsulatus* Bath and *M. trichosporium* OB3b showed a strong positive correlation to that observed in *E. coli* with R-squared values of 0.74 and 0.85, respectively (Fig. 2B and C). We measured BBa_J23104 to be the strongest promoter in all strains tested, including *E. coli*, although BBa_J23100 was originally reported as the strongest of the collection, which showed similar, but lower, strength compared to BBa_J23104 (http://parts.igem.org/Promoters/Catalog/Anderson). The difference in the activity observed here compared to prior analyses could be due to *E. coli* strain variations since we measured fluorescence in DH10B while others have used DH5α. Recently, BBa_J23119 was demonstrated to display high activity in the Gammaproteobacterial methanotroph *Methylotuvimicrobium buryatense* 5GB1C, but it was suggested that other promoters in the series may not function in methanotrophs due to a lack of measured BBa_J23112 and BBa_J23117 activity in *M. buryatense* [39]. Notably, these two promoters have limited activity in *E. coli*, and we observed low activity of BBa_J23117 in *M. capsulatus* Bath and *M. trichosporium* OB3b (Fig. 2A), but we show herein that other promoters in the Anderson series can promote transcription in diverse methanotrophs. Collectively, the BHR Anderson promoter series represents variable strength, constitutive promoters that can be used for gene expression and metabolic engineering in industrially relevant methanotrophic bacteria.

### 3.3 Generation and characterization of particulate methane monooxygenase promoter variants

**Fig. 3:**
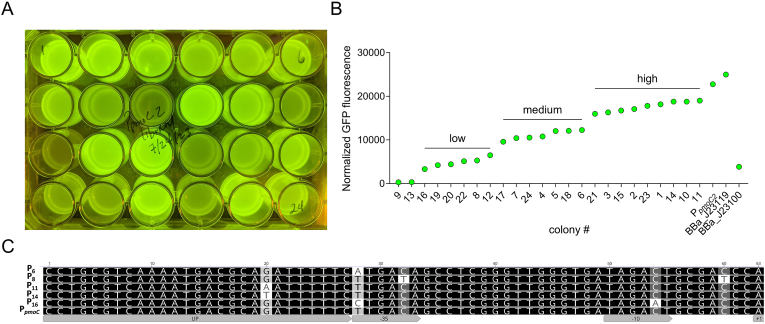
**Particulate methane monooxygenase promoter variant activity in *E. coli*.** A and B**)** Relative sfGFP fluorescence of selected particulate methane monooxygenase promoter (P_*pmoc2*_) mutagenesis library *E. coli* transformants harboring a P_*pmoc2*_-sfGFP reporter plasmid. C) Sequence alignment of P_*pmoc2*_ promoter variants with mutations in the core upstream (UP), −35, and −10 promoter elements with measured “low”, “medium”, and “high” activities.

The Anderson series promoters have mutations in the −35 and/or −10 promoter elements that decrease the DNA binding affinity of the RNA polymerase sigma factor, significantly reducing transcription initiation compared to the consensus BBa_J23119 promoter. Indeed, we measured a 10-fold difference in fluorescence between the “strongest” mutated promoter, BBa_J23104, and BBa_J23119 in *E. coli*, *M. capsulatus* Bath, and *M. trichosporium* OB3b (Fig. 2A). We sought to identify promoters with activity between BBa_J23104 and BBa_J23119 levels since promoter activity at this strength is desired for many applications. We previously showed that the *M. capsulatus* Bath particulate methane monooxygenase operon promoters (P_*pmoC1*_ and P_*pmoC2*_) are highly active in *E. coli* and display similar relative strength (P_*pmoC2*_ > P_*pmoC1*_) compared to their native activity [10]. Further, the *M. capsulatus* Bath P_*pmoC2*_ promoter exhibits similar high activity as the BBa_J23119 promoter in *E. coli*. Given the comparable activity of P_*pmoC2*_ in *E. coli* and *M. capsulatus*, we decided to use *E. coli* for rapid screening of a P_*pmoC2*_ mutant library to identify promoter variants with activity greater than the strongest Anderson BBa_J23104 variant but less than the wild-type P_*pmoC2*_ or BBa_J23119 promoters. The P_*pmoC2*_ promoter has a putative upstream (UP) RNA polymerase binding site, a −35 sequence identical to BBa_J23119, and a −10 sequence with three nucleotide differences compared to BBa_J23119 [38]. We hypothesized that mutation(s) in the P_*pmoC2*_ promoter would generate variants with the desired activity between BBa_J23104 and BBa_J23119 or wild-type P_*pmoC2*_. To test this hypothesis, we mutagenized a 150 bp P_*pmoC2*_ fragment spanning −113 through the +37 compared to the transcriptional start site [38]. The mutagenesis library was cloned into the P_*pmoC2*_ expression plasmid containing the sfGFP reporter, replacing the wild-type promoter [10]. Due to the high activity of the P_*pmoC2*_ promoter in *E. coli*, we were able to select transformants based on visual detection of sfGFP, picking 24 colonies ranging from low, medium, and high sfGFP expression compared to the wild-type P_*pmoC2*_ control (Fig. 3A)*. E*. *coli* transformant colonies harboring the wild-type P_*pmoC2*_ or high-level variant promoter expression vectors were noticeably smaller than low-level, medium-level, or no plasmid-control transformants, indicating that the high expression of sfGFP causes a fitness defect (data not shown). As expected, we measured low, medium, and high fluorescence in the transformants, which was between that determined for BBa_J23100 and BBa_J23119 (Fig. 3B).

**Fig. 4:**
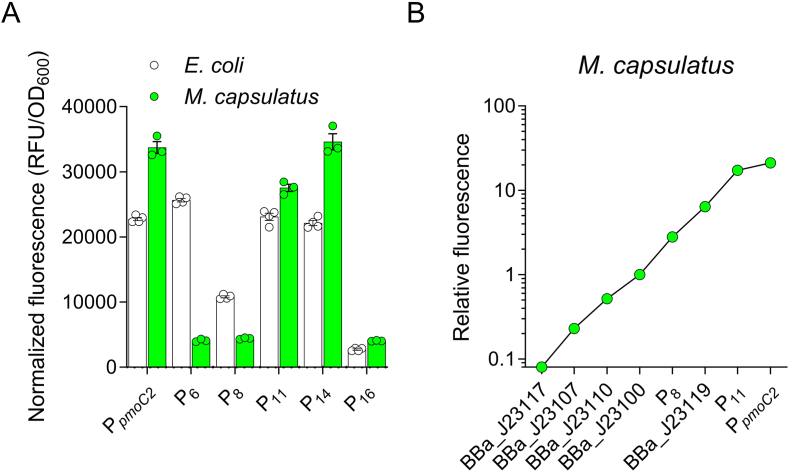
**Particulate methane monooxygenase promoter variants expand the methanotroph genetic toolbox**. A) Comparison of mutant and wild-type particulate methane monooxygenase promoter (P_*pmoC2*_) activity in *E. coli* (white bar) and *M. capsulatus* (green bar). B) The dynamic range of selected Anderson series and P_*pmoC2*_ variant promoter activity in *M. capsulatus*.

**Fig. S1:**
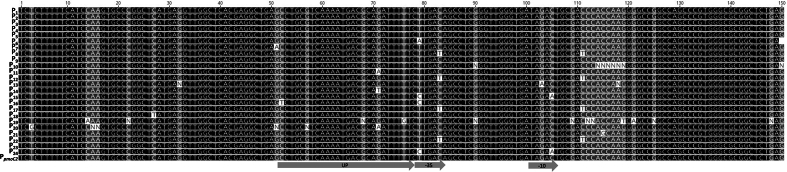
Particulate methane monooxygenase promoter variant sequences. Sequence alignment of the particulate methane monooxygenase promoter (P_*pmoc2*_) region in plasmid DNA isolated from twenty-four transformants (P_1-24_) obtained after promoter mutagenesis as described in materials and methods and shown in Fig. 3. The upstream (UP), −35, and −10 promoter elements are marked on the wild-type sequence.

Sanger sequencing of the plasmid promoter region identified mutations in the core promoter UP, −35, and −10 elements in many transformants, but not all (Supplemental Fig. S1). An A to T transversion was identified in the *sfgfp* start codon in plasmids derived from transformants 9 and 13, which exhibited no fluorescence, but other mutations outside the core promoter were not identified in the library despite mutagenesis of a larger region, indicating that additional promoter elements/binding sites outside the core promoter are not present within the mutated region. We expect that our biased colony selection process excluded other mutations outside the promoter that likely exhibited similar GFP expression as the control wild-type promoter. Many of the transformants with similar relative fluorescence had overlapping mutations, so we selected five P_*pmoC2*_ promoter variants to compare in *M. capsulatus* Bath: two “low” (P_16_, -35T to C and -9C to A; P_6_, -35T to A), one “medium” (P_8_, -31C to T and -3C to T), and two “high” (P_11_ -43G to A; P_14_ -43G to T) (Fig. 3C). *M. capsulatus* Bath transformants with wild-type P_*pmoC2*_ or P_14_ promoters showed visibly “high” sfGFP expression, but were smaller colonies compared to the other transformants one week after selection (data not shown). This small colony phenotype was comparable to the fitness defect observed in *E. coli.* Similarly, we identified transformants with no visible sfGFP expression, which was correlated to mutations in the *sfgfp* start codon (data not shown). Presumably, the cells mutate the start codon as a strategy to overcome the fitness defect associated with dedicating resources to sfGFP expression. P_6_, P_8_, and P_16_ variants showed significantly less activity compared to the wild-type promoter, highlighting mutations in the −35 and −10 regions can disrupt RNA polymerase transcription initiation (Fig. 4A). However, in disagreement with our original hypothesis, the mutations in the UP element of the P_11_ and P_14_ variants had minimal effect on promoter activity in either *E. coli* or *M. capsulatus* Bath (Fig. 4A). It is possible that other regions of the UP element could be important for enhancing RNA polymerase affinity to the promoter, but we did not identify any other UP mutations in our screen. Further, there is no experimental evidence verifying that this region identified upstream of the core −35 region is a bona fide UP element. The T to A transversion at the −35 position of the P_6_ promoter decreased promoter activity in *M. capsulatus* Bath but not in *E. coli*, underscoring potential structural differences in the RNA polymerase σ70 subunit between these bacteria (Fig. 4A). Notably, wild-type P_*pmoC2*_ promoter activity was higher in *M. capsulatus* Bath compared to the BBa_J23119 promoter (Fig. 4B). Together with the Anderson series promoters, P_*pmoc2*_ and mutant variants expand the constitutive promoter activity range to ∼2.5 orders of magnitude (Fig. 4B).

## 4 Conclusions

Targeted removal of atmospheric CH_4_ is a solution to mitigate the effects of anthropogenic climate change. Biological conversion of CH_4_ using methanotrophic bacteria can be leveraged to mitigate GHG emissions either at point sources or coupled to direct air capture technologies given their capacity to utilize CH_4_ as a carbon and energy source. Genetic engineering of these organisms will likely be required to realize the optimal utility of methanotrophs, but the currently available genetic tools are limited. Here, we have constructed and characterized a suite of constitutive promoters that exhibit variable strength in phylogenetically diverse methanotrophic bacteria. These genetic tools expand those currently available and will enable fine-tuned gene expression in methanotrophic bacteria for diverse general and applied research efforts. Notably, these promoters were assembled with BHR (pBBR and IncQ) plasmids that establish a collection of expression plasmids that have broad utility in not only methanotrophs, but other phylogenetically diverse bacteria that recognize these replicons.

## Registry of standard biological parts

The *M. capsulatus* P_*pmoc2*_ promoter variants generated during this study have been assigned the following part numbers in the iGEM registry of standard biological parts:

P6 – BBa_K4848000.

P8 – BBa_K4848001.

P11 – BBa_K4848002.

P14 – BBa_K4848003.

P16 – BBa_K4848004.

## CRediT authorship contribution statement

**Etash H. Bhat:** performed experiments, wrote the manuscript, member of the 2023 UNT iGEM team. **Jessica M. Henard:** performed experiments. **Spencer A. Lee:** conceived the project, performed experiments. **Dustin McHalffey:** performed experiments. **Mahith S. Ravulapati:** performed experiments, member of the 2023 UNT iGEM team. **Elle V. Rogers:** performed experiments, member of the 2023 UNT iGEM team. **Logan Yu:** performed experiments, member of the 2023 UNT iGEM team. **David Skiles:** performed experiments, All others edited and approved the manuscript. **Calvin A. Henard:** conceived the project, acquired funding, analyzed and graphed data, and provided overall guidance, wrote the manuscript.

## Declaration of competing interest

The authors declare that they have no known competing financial interests or personal relationships that could have appeared to influence the work reported in this paper.
